# Supplementary material for: Gene Expression of Ethanol and Acetate Metabolic Pathways in the Acinetobacter baumannii EmaSR Regulon
Source: Microorganisms. 2024 Feb 4;12(2):331. doi: 10.3390/microorganisms12020331 (PMC10891947; doi:10.3390/microorganisms12020331)
Supplement: Supplementary file 1 [file microorganisms-12-00331-s001.zip › microorganisms-2833560-supplementary.pdf]

microorganisms

Supplementary Materials

Gene Expression of Ethanol and Acetate Metabolic Pathways in the *Acinetobacter baumannii* EmaSR Regulon

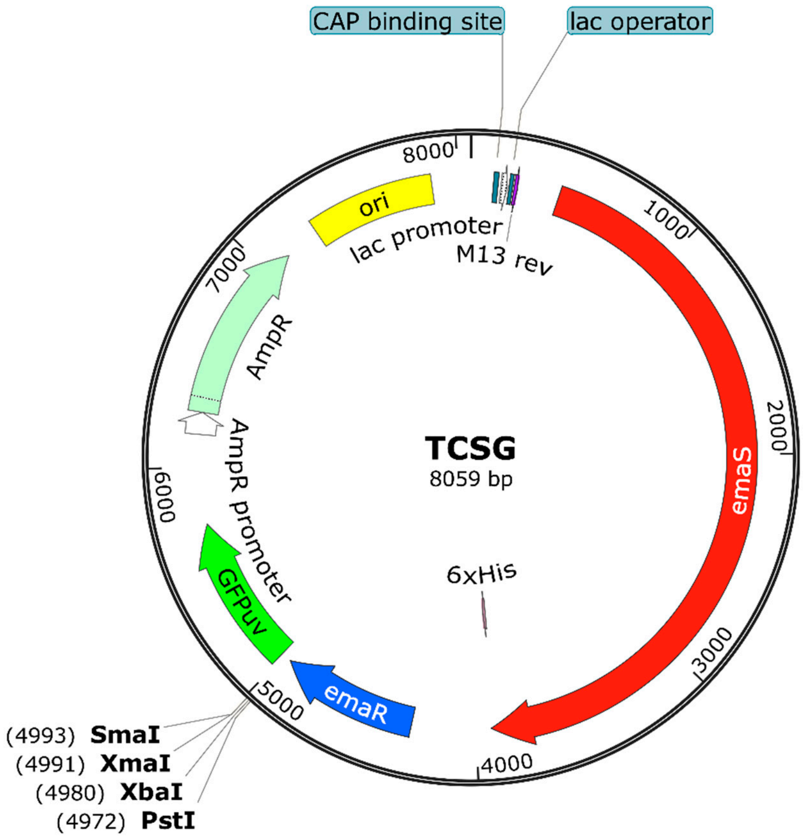

Figure S1. Map of the TCSG plasmid indicating genes and restriction enzyme sites. The origin of replication (*ori*, yellow rectangle), *emaS* (red arrow), *emaR* (blue arrow), *gfpuv* (bright green, representing GFPuv), and the ampicillin resistance gene Amp^r (light green, denoted as AmpR) have been indicated.

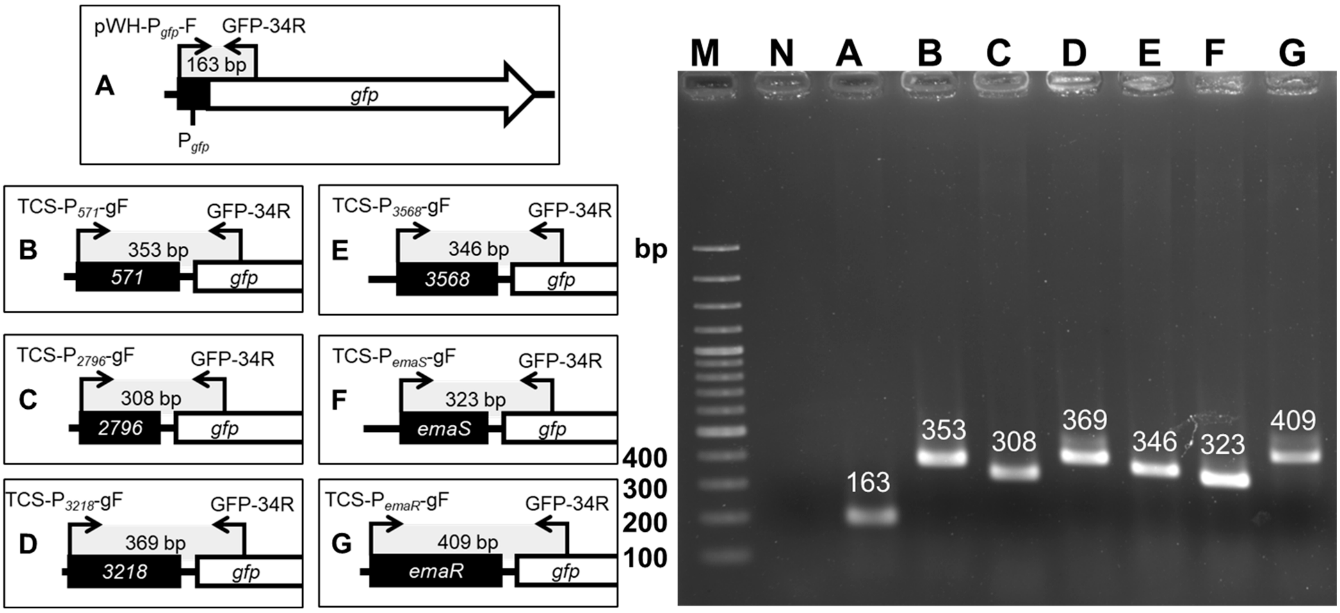

Figure S2. Confirmation of *E. coli* transformation strains carrying promoter-TCSG. Gene maps of (A) TCSG, (B) TCS-P$_{571}$G, (C) TCS-P$_{2796}$G, (D) TCS-P$_{3218}$G, (E) TCS-P$_{3568}$G, (F) TCS-P$_{emaS}$G, and (G) TCS-P$_{emaR}$G are presented, along with the complementary positions of primers used for plasmid construction and the predicted sizes of amplified DNA fragments. The image at right shows the electrophoresis results of colony PCR products derived from the target plasmids. M represents a 100 bp DNA marker, while N is a negative control group without template. Columns A-G indicate results from transformants containing the plasmids shown in panels A-G, with numbers representing length in base pairs (bp) of the amplified DNA fragments.

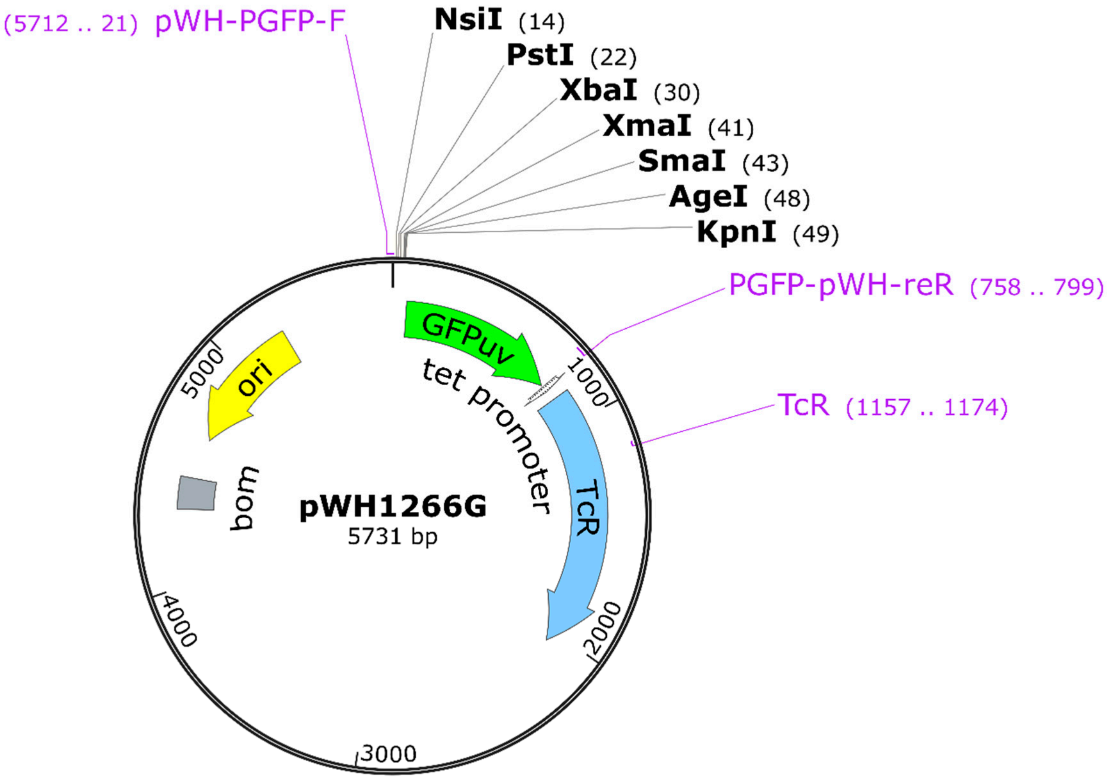

Figure S3. Map of the pWH1266G plasmid. Plasmid pWH1266G was constructed by Gibson assembly. The yellow arrow signifies the origin of replication (*ori*) in *E. coli*, the light blue arrow represents the tetracycline resistance gene (TcR), and the lime green arrow corresponds to the green fluorescent protein gene (GFPuv).

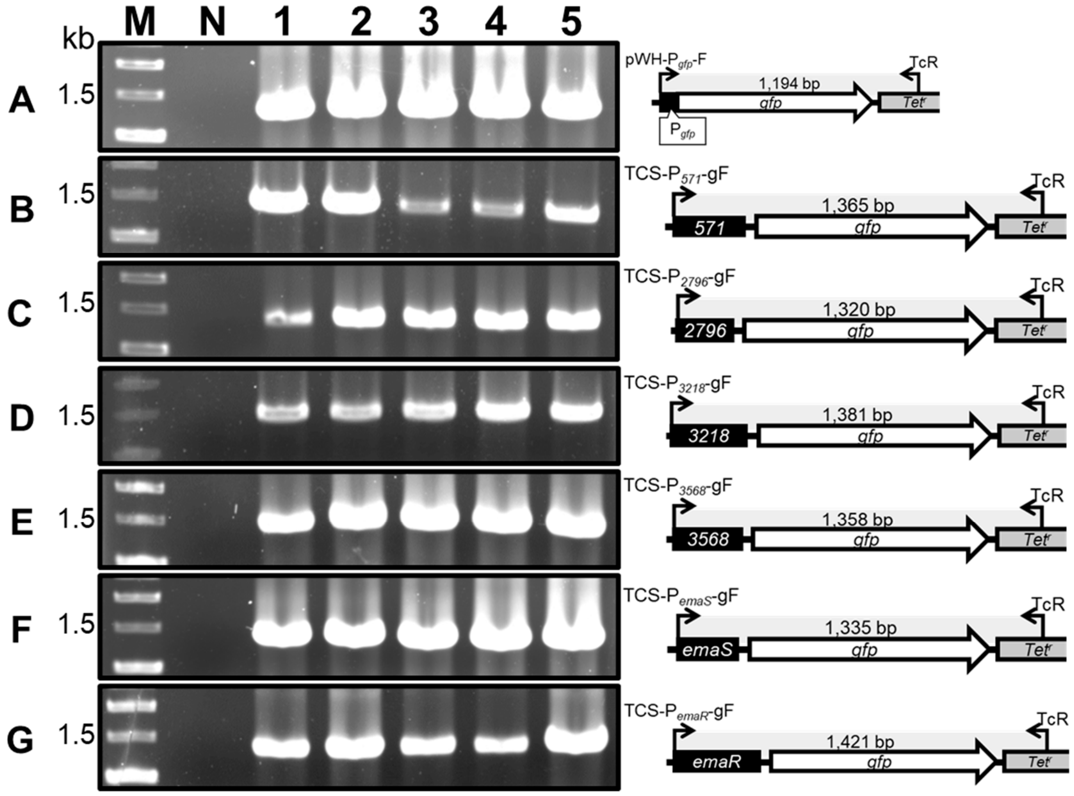

Figure S4. Confirmation of *E. coli* transformation strains carrying pWH1666-derived reporter plasmids. PCR product analysis results for reporter plasmids (A) pWH1266G, (B) pWH-P$_{571}$G, (C) pWH-P$_{2796}$G, (D) pWH-P$_{3218}$G, (E) pWH-P$_{3568}$G, (F) pWH-P$_{emaS}$G, and (G) pWH-P$_{emaR}$G, which were successfully constructed in (1) *E. coli* and then introduced by electroporation into various *A. baumannii* strains, including (2) wild-type, (3) *emaS*, (4) *emaR*, and (5) *emaSR*. Negative control samples (N) that lack a template were included in each analysis. M represents a 100 bp DNA marker. Panels at right present the gene maps of the corresponding reporter plasmids, along with the positions of the PCR primers used for plasmid construction, as well as the expected sizes of the amplified DNA fragments.

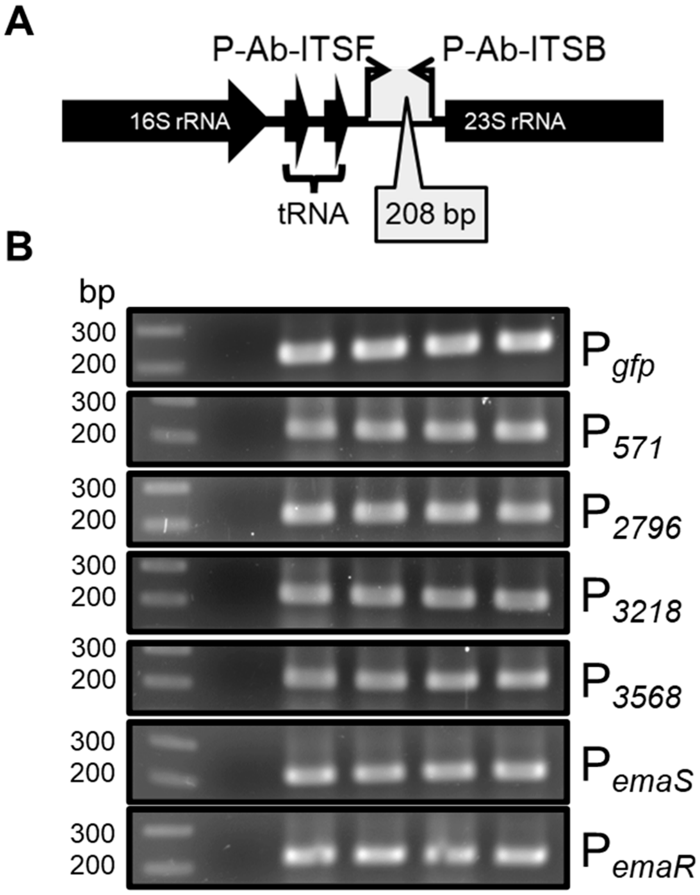

Figure S5. PCR product analysis by 2% agarose gel electrophoresis. (A) Gene map indicating the names of primer pairs used to amplify the intergenic spacer (ITS) between 16S and 23S rRNA and their complementary positions on the chromosome of *A. baumannii*. The predicted length (in base pairs, bp) of the amplified DNA fragment is indicated. (B) ITS fragments were amplified in (1) *A. baumannii* wild-type, (2) Δ*emaS*, (3) Δ*emaR*, and (4) Δ*emaSR*, which were obtained separately after transformation of pWH1266G (P_{gfp}), pWH-P_{571}G (P_{571}), pWH-P_{2796}G (P_{2796}), pWH-P_{3218}G (P_{3218}), pWH-P_{3568}G (P_{3568}), pWH-P_{emaS}G (P_{emaS}), and pWH-P_{emaR}G (P_{emaR}) by electroporation. M represents a 100 bp DNA marker, while N is a negative control group without template.

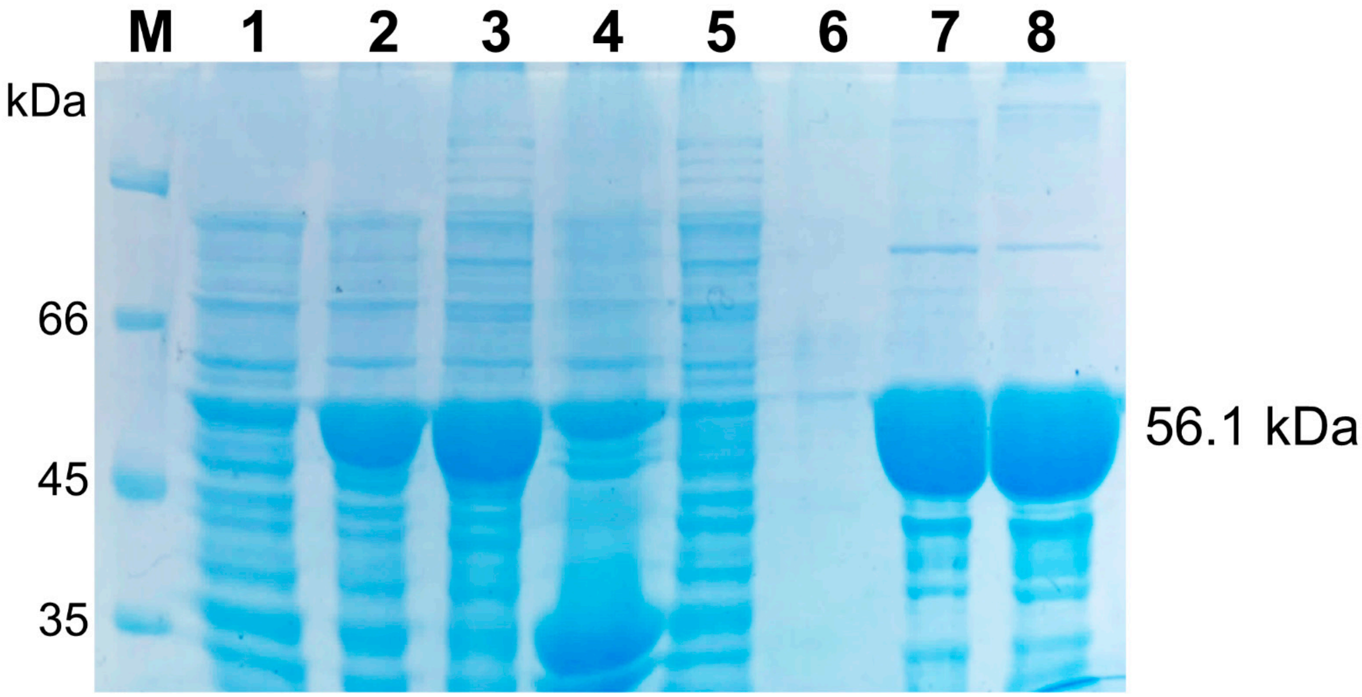

Figure S6. Analysis of expressed and purified DJ41_2796 protein by 10% SDS-PAGE. M represents the protein molecular weight marker (units in kiloDaltons, kDa); Lane 1 represents the sample before induction with 0.5 mM isopropyl ß-D-1-thiogalactopyranoside (IPTG); Lane 2 represents the sample after 3 hours of IPTG induction for DJ41_2796 expression; Lanes 3 and 4 respectively represent the supernatant and pellet after bacterial cell disruption; Lane 5 represents the sample collected after passing the supernatant through a nickel affinity column; Lane 6 represents the sample collected after washing the column with 6-13% elute buffer; and Lanes 7 and 8 represent samples collected after passing 100% elute buffer containing the target protein DJ41_2796 through the column. The numbers indicated on the right represent the expected molecular weights of DJ41_2796.

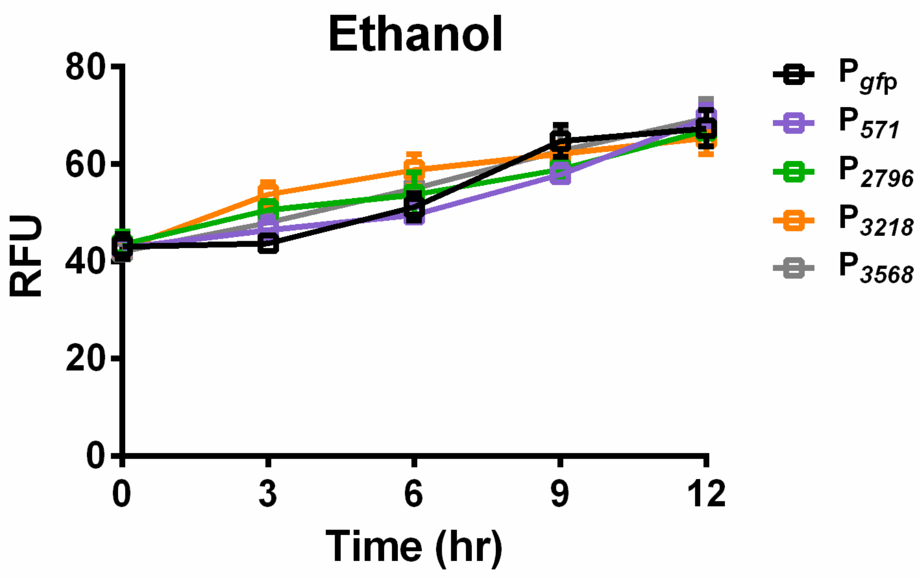

Figure S7. Mean expression levels of EmaSR regulons in *E. coli* cultured in LB containing 1% ethanol. *E. coli* carrying reporter plasmids with TCSG (black), TCSG-P_{571} (purple), TCSG-P_{2796} (green), TCSG-P_{3218} (orange), and TCSG-P_{3568} (gray) were cultured in LB medium containing 1% ethanol, with initiation OD_{600} of 0.1. The x-axis represents time (hours), and the y-axis shows the relative fluorescence units (RFU). Results were derived from three independent experiments.

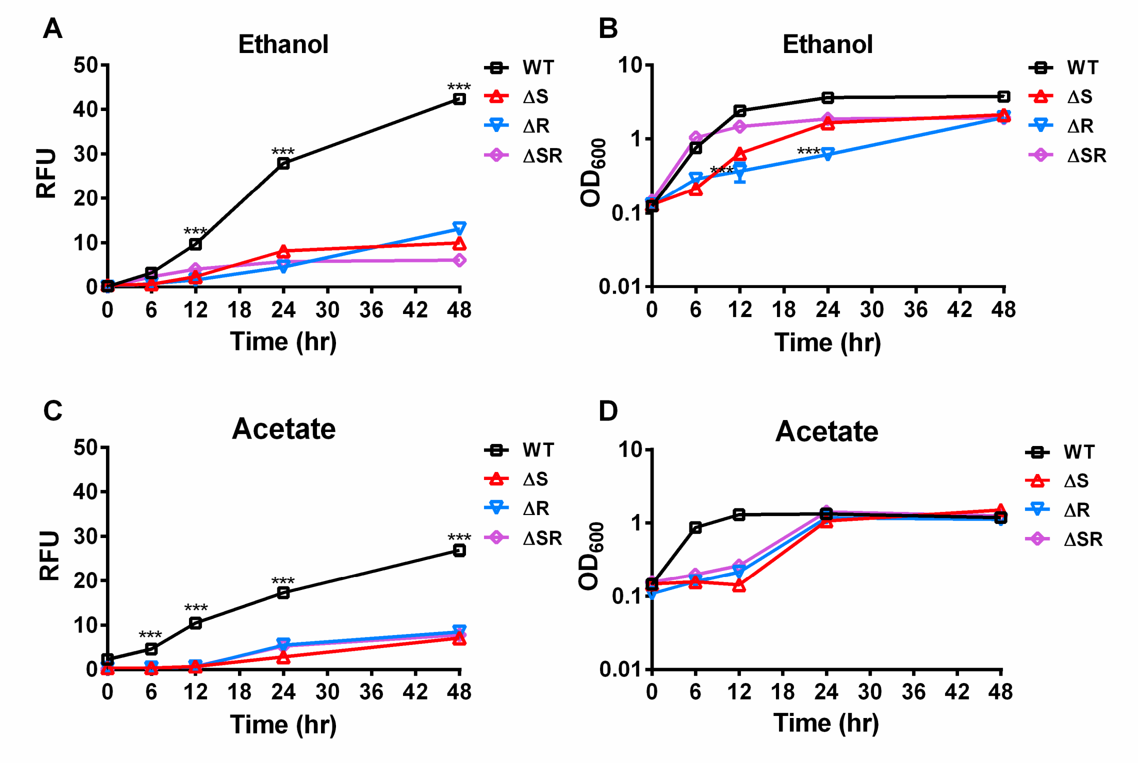

Figure S8. Mean P2796 fluorescence expression levels in different *A. baumannii* strains cultured in low-concentration ethanol and acetate. Changes in fluorescence levels over 48 hours were observed in wild-type (WT, black square), Δ*emaS* (ΔS, red triangle), Δ*emaR* (ΔR, blue inverted triangle), and Δ*emaSR* (ΔSR, purple diamond) strains of *A. baumannii*, each carrying the pWH-P2796G reporter plasmid. Strains were cultured with (**A, B**) 0.5% ethanol or (**C, D**) 20 mM acetate, with fluorescence expression levels (**A, C**) and growth curves (**B, D**) indicated. ***p < 0.0001, multiple t-test. RFU, relative fluorescence units. OD, optical density. Data were derived from at least three independent experiments.

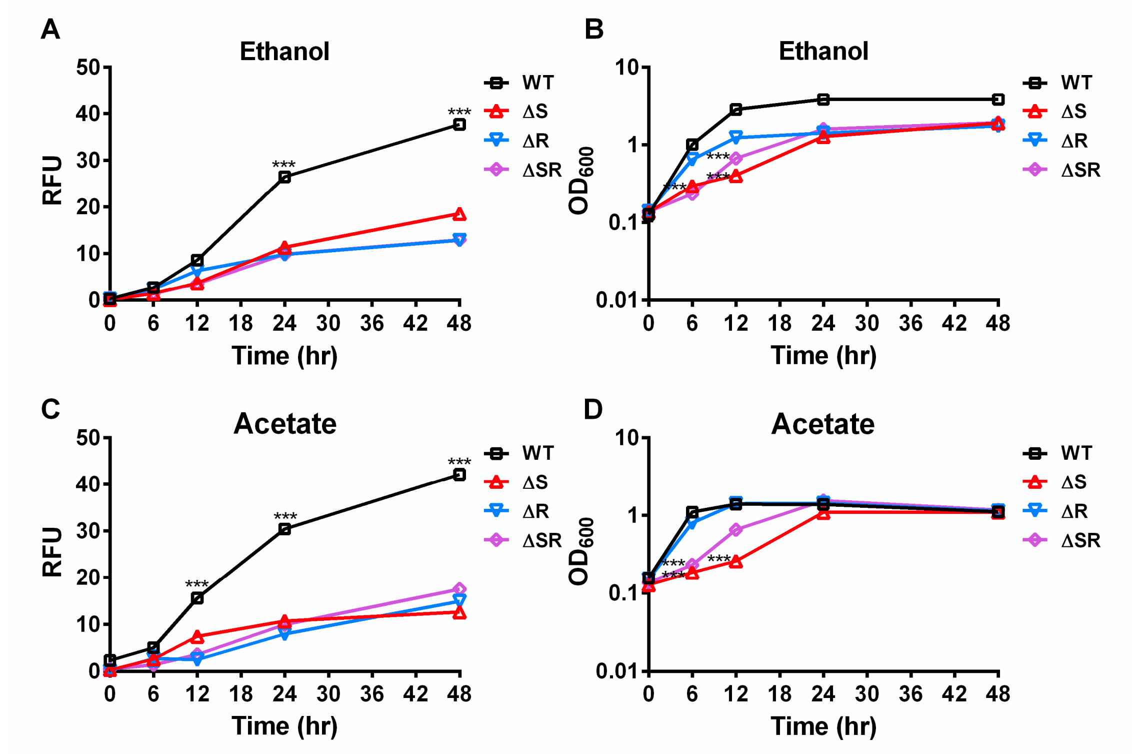

Figure S9. Mean P_{3218} fluorescence expression levels in different *A. baumannii* strains cultured in low-concentration ethanol and acetate. Changes in fluorescence levels over 48 hours were observed in wild-type (WT, black square), Δ*emaS* (ΔS, red triangle), Δ*emaR* (ΔR, blue inverted triangle), and Δ*emaSR* (ΔSR, purple diamond) strains of *A. baumannii*, each carrying the pWH-P_{3218}G reporter plasmid. Strains were cultured with (**A, B**) 0.5% ethanol or (**C, D**) 20 mM acetate, with fluorescence expression levels (**A, C**) and growth curves (**B, D**) indicated. ***p < 0.0001, multiple t-test. RFU, relative fluorescence units. OD, optical density. Data were derived from at least three independent experiments.

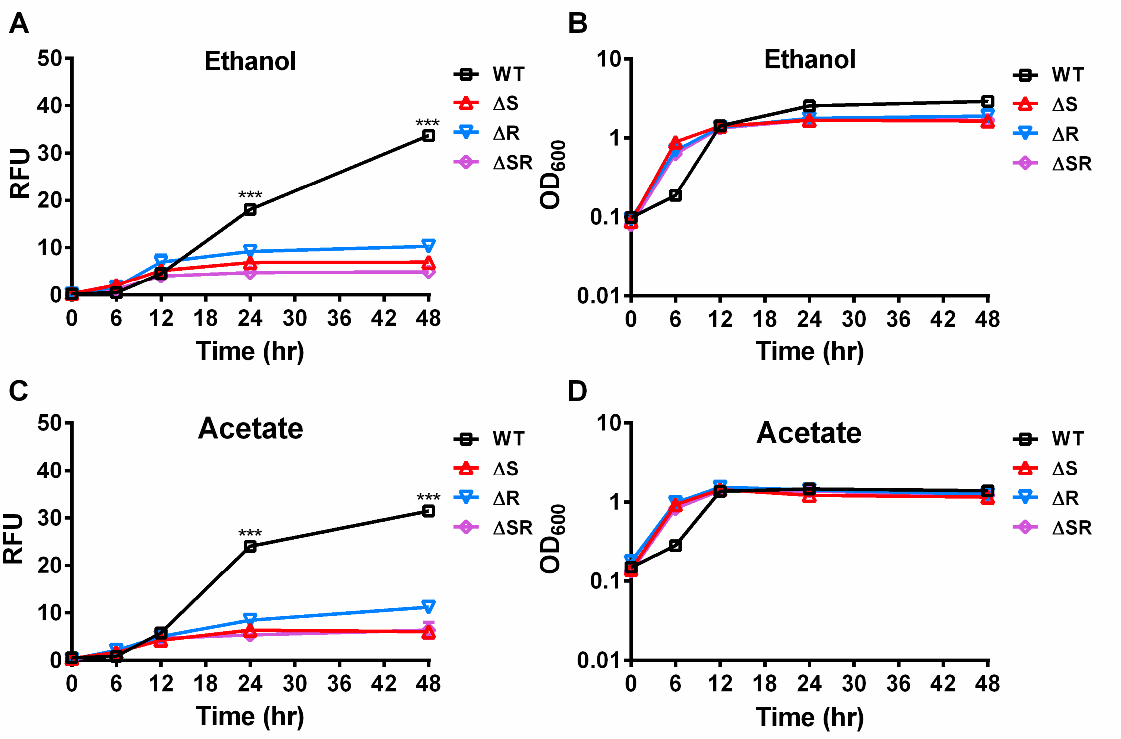

Figure S10. Mean P_{emaR} fluorescence expression levels in different *A. baumannii* strains cultured in low-concentration ethanol and acetate. Changes in fluorescence levels over 48 hours were observed in wild-type (WT, black square), Δ*emaS* (ΔS, red triangle), Δ*emaR* (ΔR, blue inverted triangle), and Δ*emaSR* (ΔSR, purple diamond) strains of *A. baumannii*, each carrying the pWH-P_{emaR}G reporter plasmid. Strains were cultured with (**A, B**) 0.5% ethanol or (**C, D**) 20 mM acetate, with fluorescence expression levels (**A, C**) and growth curves (**B, D**) indicated. ***p < 0.0001, multiple t-test. RFU, relative fluorescence units. OD, optical density. Data were derived from at least three independent experiments.

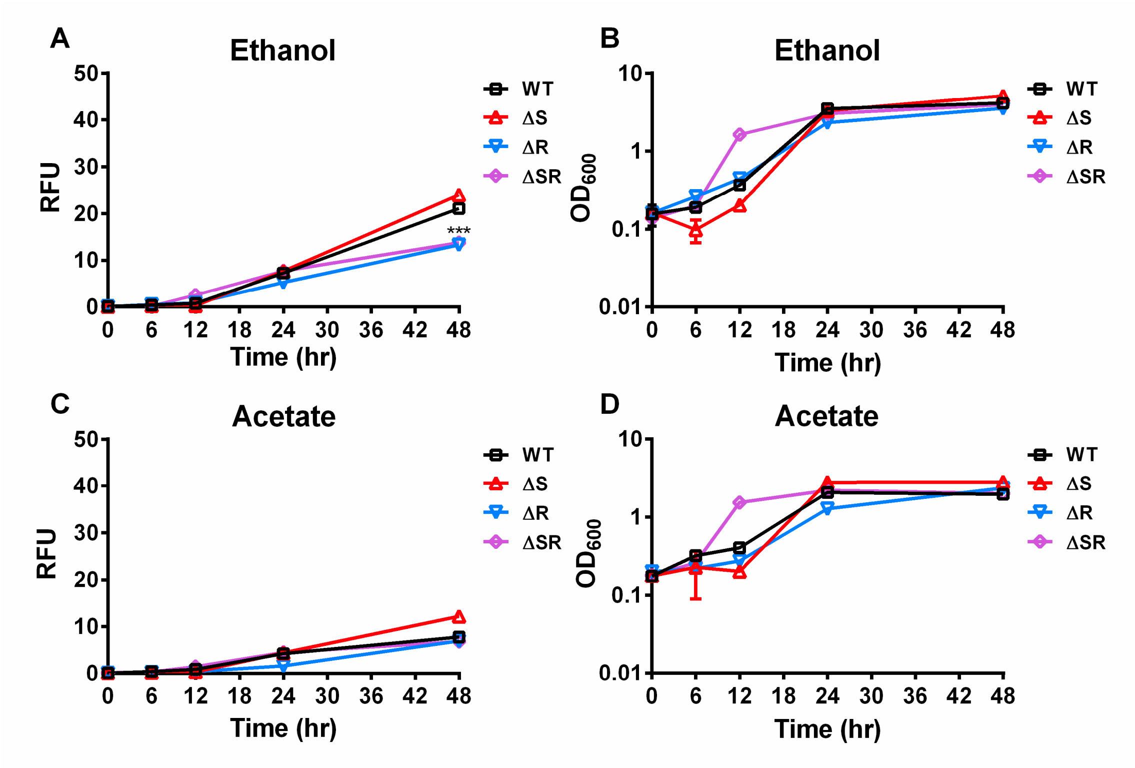

Figure S11. Mean P*emaS* fluorescence expression levels in different *A. baumannii* strains cultured in low-concentration ethanol and acetate. Changes in fluorescence levels over 48 hours were observed in wild-type (WT, black square), Δ*emaS* (ΔS, red triangle), Δ*emaR* (ΔR, blue inverted triangle), and Δ*emaSR* (ΔSR, purple diamond) strains of *A. baumannii*, each carrying the pWH-P*3218*G reporter plasmid. Strains were cultured with (**A, B**) 0.5% ethanol or (**C, D**) 20 mM acetate, with fluorescence expression levels (**A, C**) and growth curves (**B, D**) indicated. ***p < 0.0001, multiple t-test. RFU, relative fluorescence units. OD, optical density. Data were derived from at least three independent experiments.
